# Conformational Distortions of the Red Blood Cell Spectrin Matrix Nanostructure in Response to Temperature Changes *In Vitro*

**DOI:** 10.1155/2019/8218912

**Published:** 2019-05-06

**Authors:** Elena Kozlova, Aleksandr Chernysh, Viktoria Sergunova, Ekaterina Manchenko, Viktor Moroz, Aleksandr Kozlov

**Affiliations:** ^1^V.A. Negovsky Research Institute of General Reanimatology, Federal Research and Clinical Center of Intensive Care Medicine and Rehabilitology, 107031, 25 Petrovka St., Build. 2, Moscow, Russia; ^2^Sechenov First Moscow State Medical University (Sechenov University), 119991, 2-4 Bolshaya Pirogovskaya St, Moscow, Russia

## Abstract

The spectrin matrix is a structural element of red blood cells (RBCs). As such, it affects RBC morphology, membrane deformability, nanostructure, stiffness, and, ultimately, the rheological properties of blood. However, little is known about how temperature affects the spectrin matrix. In this study, the nanostructure of the spectrin network was recorded by atomic force microscopy. We describe how the nanostructure of the RBC spectrin matrix changes from a regular network to a chaotic pattern following an increase in temperature from 20 to 50°C. At 20–37°С, the spectrin network formed a regular structure with dimensions of typically 150 ± 60 nm. At 42–43°С, 83% of the spectrin network assumed an irregular structure. Finally, at 49–50°С the chaotic pattern was observed, and no quantitative estimates of the spectrin structure's parameters could be made. These results can be useful for biophysical studies on the destruction of the spectrin network under pathological conditions, as well as for investigating cell morphology and blood rheology in different diseases.

## 1. Introduction

Red blood cell (RBC) morphology, membrane deformability, and nanostructure are largely related to the structure of the cytoskeleton underlying the lipid bilayer [1–3]. The spectrin matrix is thought to be a critical structural component of RBCs [4].

Temperature represents an important factor affecting the functioning of biological objects. An increase in temperature causes conformational distortions of the RBC spectrin matrix [5] and changes the rheological properties of blood [6].

Various methods have been applied to study the RBC spectrin matrix [7–9]. Recently, atomic force microscopy (AFM) has been used to visualise blood cells and study the properties of their membranes. AFM is particularly effective for studying the nanostructure of biological objects up to several nanometres in size [10–22].

External factors, such as ionizing radiation [23], drugs [24, 25], or long-term storage of packed RBCs [26], as well as genetic mutations [27] can damage both protein and lipid components of the membrane. This is then followed by damage to the spectrin matrix. Understanding how the RBC spectrin network behaves under different physical and chemical insults remains an important scientific and clinical challenge.

The purpose of the present study was to apply AFM to investigate the influence of temperature on conformational distortions of the RBC spectrin matrix nanostructure *in vitro*.

## 2. Materials and Methods

### 2.1. Experimental Setup

Experimental work was carried out according to the following scheme:

1. Development of the ghost preparation method for spectrin matrix identification
2. Acquisition of spectrin network AFM images
3. Investigation of the influence of the physical factor (temperature) on cells
4. Quantitative assessment of spectrin matrix characteristics based on the effect of the above factor

### 2.2. Preparation of Blood Samples

Blood withdrawal (150 *μ*L) into ethylenediaminetetraacetic acid- (EDTA-) containing microvettes (Sarstedt AG & Co., Germany) was carried out during a prophylactic examination on eight volunteers (25–35 years old, four women and four men). Informed consent was obtained from each donor. All experiments were carried out in accordance with guidelines and regulations of the Federal Research and Clinical Centre of Intensive Care Medicine and Rehabilitology, V.A. Negovsky Scientific Research Institute of General Reanimatology, Moscow, Russian Federation. All experimental protocols were approved by this Institute.

### 2.3. Temperature Adjustment

Microvettes containing blood samples were put into a hot-air thermostat TC-1/80 (SKTB SPU, Russian Federation) and incubated for 30 min at a given temperature. The temperature ranges were as follows: 20°С, 36–37°С, 39–40°С, 42–43°С, and 49–50°С.

### 2.4. Formation of the Spectrin Matrix on the Surface of RBC Ghosts

For the sake of clarity, we defined the term RBC ghost as the membrane and cytoskeletal elements of the RBC devoid of cytoplasmic contents. In our experiments, lipid fractions were removed from the RBC ghosts and the spectrin matrix appeared on the surface.

**Figure 1:**
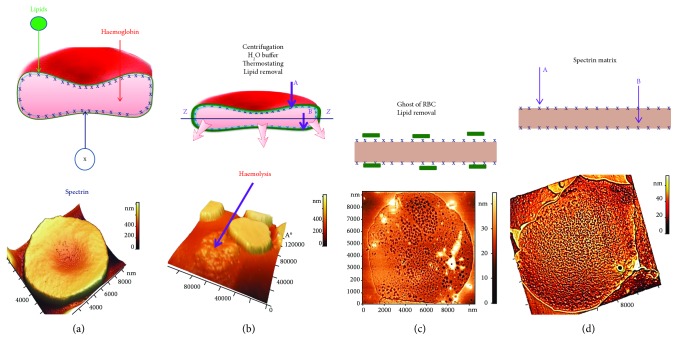
Experimental setup for detecting the spectrin matrix: schematic representation and AFM images. (a) RBC prior to treatment, (b) haemolysis and partial removal of lipids, (c) formation of RBC ghosts and lipid removal, and (d) exposure of the spectrin matrix on the ghosts' surface.

Figure 1 illustrates the formation of RBC ghosts and exposure of spectrin matrix. Cells were first subjected to haemolysis (Figure 1(a)), whereby 150 *μ*L of fresh blood was added to 500 *μ*L hypotonic solution consisting of one part 0.9% NaCl (Kelun, Kazpharm, Kazakhstan) and nine parts of distilled water. This initiated RBC haemolysis (Figure 1(b)). The resulting suspension was centrifuged in an Eppendorf tube (Ningbo Greetmed Medical Instruments Co., China) at 3,000 rpm for 5 min. The supernatant was removed, and a pellet of 75 *μ*L was left in the tube. Then, 300 *μ*L of distilled water was added to the suspension to continue haemolysis and the suspension was mixed. The resulting lysate was centrifuged at 500 rpm for 5 min. After a soft centrifugation, the Eppendorf tube with the suspension was left in the refrigerator at 4°C for 30 min and then at 20°C for 10 min to continue with haemolysis. The Eppendorf tube containing the ghost suspension was finally centrifuged at 3,000 rpm for 5 min. Once the supernatant was removed, a pellet of 75 *μ*L was left at the bottom of the tube and 10 *μ*L of it was placed on a glass slide. Smears of RBC ghosts' monolayers were prepared using a device-assisted V-Sampler (Vision Hema, Austria). Given the progressive removal of lipids during haemolysis (Figures 1(b) and 1(c)), the spectrin matrix was now exposed in RBC ghost smears (Figure 1(d)).

The use of RBC ghosts to expose the spectrin matrix on the membrane surface has been reported previously [18]. Here, only physical means were applied to achieve spectrin matrix exposure: osmosis (0.9% NaCl and distilled H_2_O), centrifugal force (3,000 rpm), and temperature (20°C and 4°C). We did not use any chemical agents for preparation of RBC ghosts—neither glutaraldehyde nor triton, etc., in order to avoid additional chemical influence on the protein-lipid structures of the membrane.

**Figure 2:**
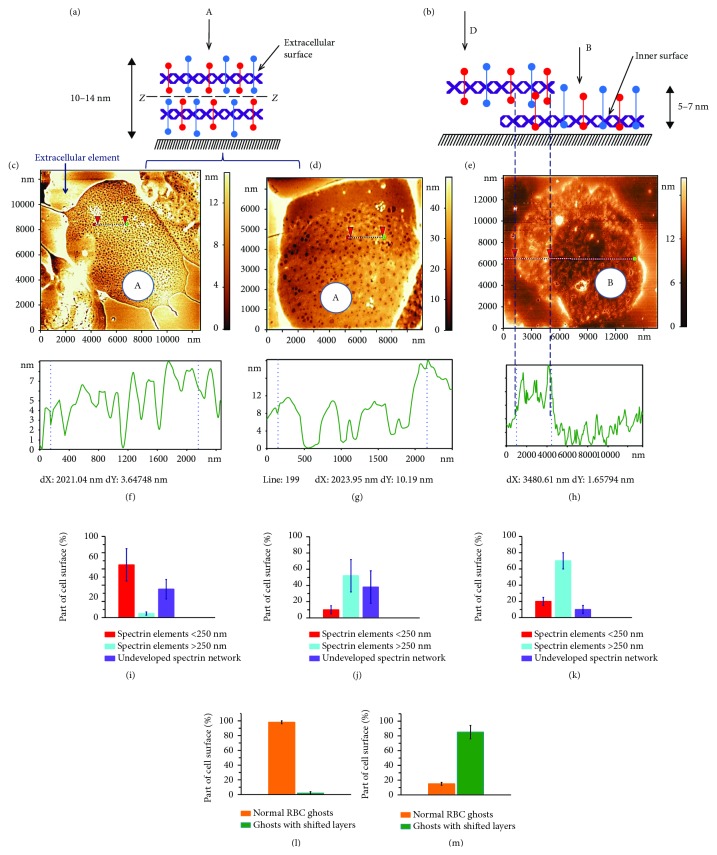
Spectrin matrix. (a) Schematic representation of the extracellular membrane surface (arrow A), a dashed line (ZZ) indicates the centre of the membrane. (b) Schematic representation of the inner membrane surface (arrow B) and shifted layer (arrow D). (c) AFM 2D image of the RBC spectrin matrix, 12 × 12 *μ*m^2^, obtained with optimised preparation parameters: 300 *μ*L distilled H_2_O and centrifugation at 3,000 rpm. (d) AFM 2D image of the RBC spectrin matrix, 8 × 12 *μ*m^2^, obtained with 500 *μ*L distilled H_2_O and centrifugation at 3,000 rpm. (e) AFM 2D image of the RBC spectrin matrix on the cytoplasmic membrane surface, 15 × 15 *μ*m^2^, obtained with 300 *μ*L distilled H_2_O and centrifugation at 12,000 rpm. (f–h) Height profiles of the corresponding sections in (c–e). (i–k) Percentage of spectrin network surfaces containing elements <250 nm and > 250 nm, as well as with an undeveloped spectrin network as determined in (c–e, m). (l–m) Percentage of spectrin network on the extracellular membrane surface and with removed or shifted layer: (l) corresponds to (e), and (m) corresponds to (c, d). The dashed line from (b) to (e) delineates the extracellular and inner membrane surfaces. Experimental data are shown as mean ± SD.

The extracellular (outer surface) and cytoplasmic (inner surface) structures of the plasma membrane can be studied by various methods [18, 28]. The spectrin matrix on the extracellular surface of the membrane is shown schematically in Figures 1(b) and 1(d) (arrow A) and Figure 2(a) (arrow A). The spectrin matrix on the cytoplasmic surface of the membrane is shown schematically in Figures 1(b) and 1(d) (arrow B) and Figure 2(b) (arrow B). The structure of the spectrin matrix on the inner side of the membrane can be observed following a shift in the ZZ plane (Figures 1(b), 2(a), and 2(b)).

If the RBC ghost maintained two complete surfaces after all the preparation steps, then the spectrin matrix on the extracellular membrane surface could be visualised (A, Figures 2(c) and 2(d)). Conversely, if the upper layer was partially removed or shifted during preparation (arrow D, Figure 2(b)), then the inner surface appeared (arrow B, Figure 2(b); B, Figure 2(e)).

Figure 2(c) shows an example of an AFM image of a spectrin matrix obtained on a control blood sample not subjected to any external factor. The typical size of the spectrin network was *S* = 150 ± 60 nm. Such a basic network was observed in 65 ± 20% of the extracellular surfaces of RBC ghosts. In 30 ± 12% of RBC ghosts' surfaces, the network could not be detected. The remaining 5 ± 2% of RBC ghosts' surfaces had a spectrin network whose dimensions were typically >250 nm (Figure 2(i)). Only 2% of the ghosts' surfaces presented shifted layers (Figure 2(l)).

Observation of the spectrin matrix and its structural characteristics depends on preparation conditions. Accordingly, an increase in the volume of distilled water during the preparation of the spectrin matrix by a factor of 1.5 (up to 500 *μ*L) increased by ten times the number of large spectrin networks (Figures 2(d), 2(g) and 2(j)). An increase in the rotational rate of RBC ghosts to 12,000 rpm led to the rupture of spectrin filaments (Figures 2(e), 2(h), 2(k), and 2(m)) in comparison with the original procedure (Figures 2(c), 2(f), 2(i), and 2(l)) and to 85% of the ghosts having shifted layers (Figure 2(m)). A representative AFM image of the spectrin matrix on the cytoplasmic membrane surface prepared using high-speed centrifugation is shown in Figures 2(e) and 2(h).

### 2.5. Atomic Force Microscopy

AFM was used to visualise the spectrin matrix and to quantify its characteristics [18, 20]. We used the NTEGRA Prima AFM instrument (NT-MDT Co., Russian Federation) under the semi-contact mode. Cantilevers PPP-NCSTR-10 (Nanosensors, Switzerland) with a force constant of 5 N/m, tip radius of 10–15 nm, and resonance frequency of 76–263 kHz were used. We employed also cantilevers NSG01 (TipsNano, Russian Federation) with a force constant of 5 N/m, tip curvature radius of 10 nm, and resonance frequency of 87–230 kHz. There were 512 or 1024 scanning points within each line of image.

We scanned monolayers of RBCs and RBC ghosts. The scanning fields were 100 × 100 *μ*m^2^, 40 × 40 *μ*m^2^, or 10 × 10 *μ*m^2^. To study the spectrin matrix nanostructure, a membrane surface area of 3 × 3 *μ*m^2^ was scanned. We analysed 2D and 3D images, as well as the corresponding profiles. For image processing and quantitative assessment of their parameters, we employed the instrument's own NT-MDT SPM software.

### 2.6. Statistical Analysis

**Figure 3:**
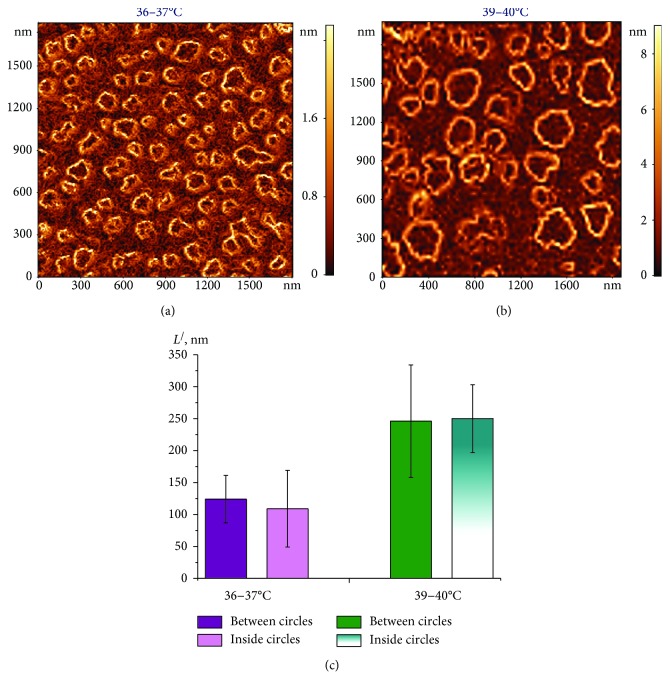
AFM 2D image of the *grad* *h* (*x*, *y*) distribution in the spectrin matrix, 2000 × 2000 nm^2^. (a) At 36–37°С; (b) at 39–40°C; (c) histogram of distance *L*^/^ (nm) between circles and inside (diameter), as determined in (a) and (b). Experimental data are shown as mean ± SD.

The following groups of RBCs from donors were analysed: control 20°С (eight donors), 36–37°С (four donors), 39–40°С (four donors), 42– 43°С (four donors), and 49– 50°С (four donors). The experiments were repeated three times. In each experiment, the corresponding sample (smear) of RBC ghosts was made, amounting to 24 RBC ghost samples for the control and 48 for the different temperature ranges. In total, we obtained 81 samples. AFM images of 100 × 100 *μ*m^2^ and 40 × 40 *μ*m^2^ were acquired for each sample and ten RBC ghosts were selected from each of them for quantitative estimation. This yielded a total of 810 RBC ghosts. To estimate nanostructure characteristics, 50 values of sizes (*L*, *S*) and height (*h*) per RBC ghost were measured and taken into account. For each condition, 1800 characteristics were estimated. Statistical analysis of experimental data was performed using OriginPro (OriginLab Corporation, Northampton, Massachusetts, USA). Data represent mean ± standard deviation (SD). Error bars in Figures 2(i)–2(m), and 3(c) represent the SD.

## 3. Results

### 3.1. Local Nanodefects Are Observed in the Spectrin Network

**Figure 4:**
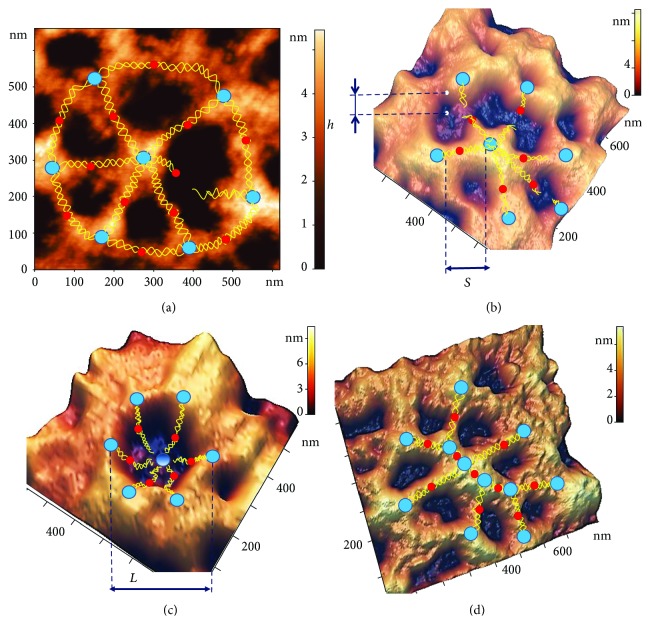
Examples of typical local nanodefects in the spectrin network. (a) AFM 2D image, 660 × 660 nm^2^, of a basic spectrin matrix element, with a broken ankyrin (red) connection. (b) AFM 3D image, 700 × 700 nm^2^, of a spectrin network element with three distorted ankyrin connections. (c) AFM 3D image, 600 × 600 nm^2^, of a spectrin matrix element, in which the connection between six spectrin filaments (yellow lines) and an actin complex (blue) is broken, resulting in the formation of a local topological crater-like dip. (d) AFM 3D image, 800 × 800 nm^2^, of a spectrin network element showing clustering of protein complexes.

Figure 4 shows some examples of typical defects in spectrin network nanostructure observed during our experiments on the influence of temperature on blood. Normally, each actin complex (blue marker, Figure 4) of the spectrin matrix comprised six spectrin filaments (yellow lines). However, as shown in Figure 4(a), one connection could occasionally be broken.

**Figure 5:**
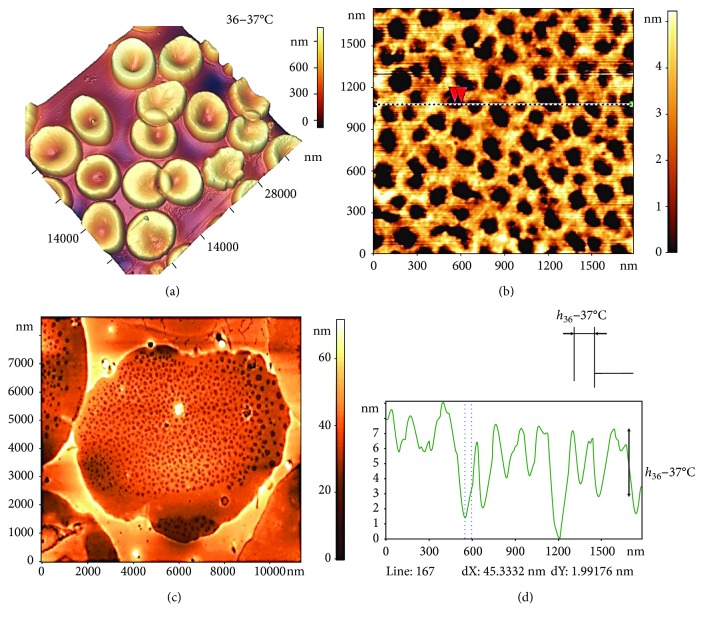
Influence of temperature at 36–37°С on spectrin matrix nanostructure. (a) AFM 3D image showing RBC morphology, 38 × 38 *μ*m^2^. (b) AFM 2D image of the RBC spectrin matrix, 8 × 8 *μ*m^2^. (c) AFM 2D image of a fragment of the spectrin matrix nanostructure, 1500 × 1500 nm^2^. (d) Height profile along the line indicated in (c), and red arrows correspond to dashed lines on the profile.

**Figure 6:**
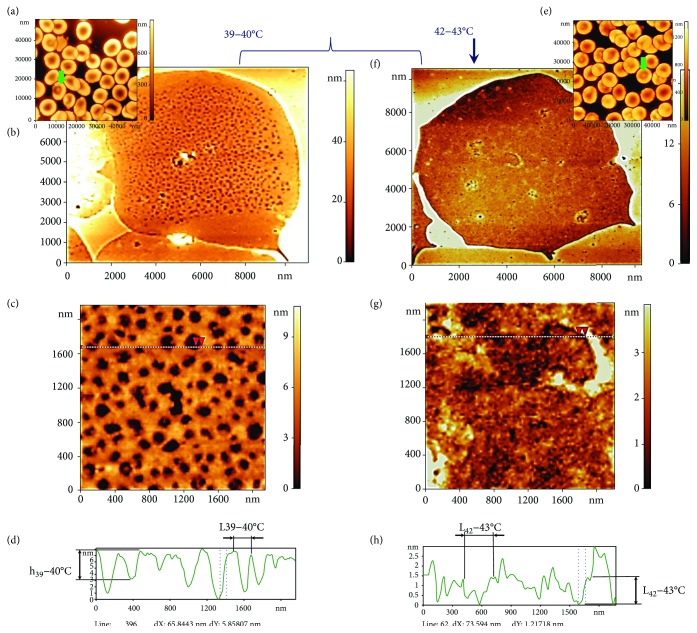
Influence of temperature at 39–40°С and 42–43°С on spectrin matrix nanostructure. (a, e) AFM 3D images of RBC morphology, 38 × 38 *μ*m^2^ and 60 × 60 *μ*m^2^. (b, f) AFM 2D images of the spectrin matrix, 12 × 12 *μ*m^2^. (c, g) AFM 2D images of a fragment of the spectrin matrix nanostructure, 2000 × 2000 nm^2^. (d) Height profile along the lines indicated in (c, h, g). Red arrows correspond to dashed lines on the profile. The curly bracket unites possible variants of RBC ghosts and spectrin matrix at 39–40°С. The vertical arrow shows a ghost RBC variant and the spectrin matrix at 42–43°С. Ensemble 1 of RBC ghosts corresponds to (b–d). Ensemble 2 of RBC ghosts corresponds to (f–h).

**Figure 7:**
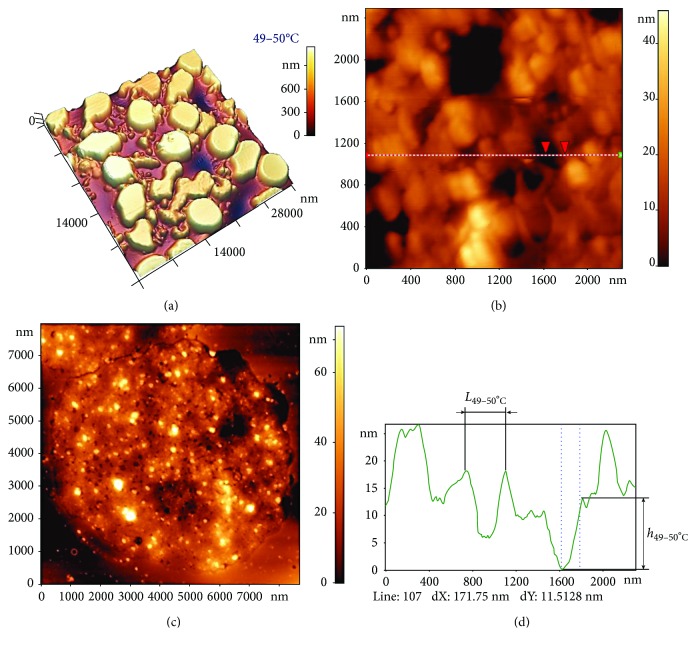
Influence of temperature at 49–50°С on the spectrin matrix nanostructure. (a) AFM 3D image showing RBC morphology, 38 × 38 *μ*m^2^. (b) AFM 2D image of the distorted spectrin network, 8 × 8 *μ*m^2^. (c) AFM 2D images of a fragment of the distorted spectrin matrix nanostructure (chaotic pattern), 2300 × 2300 nm^2^. (d) Height profile along the line indicated in (c). Red arrows correspond to dashed lines on the profile.

Based on AFM images, the characteristic length *S* of spectrin filaments was estimated as the end-to-end distance between actin nodes, whereas the height *h* of the nanostructures was determined by the elevation difference between the edge of the hole and its deepest point (Figure 4(b)). The size *L* of holes in the spectrin matrix was estimated by the surface profile (Figures 4(c), 5(c), 5(d), 6(c), 6(d), 6(g), 6(h), 7(c), and 7(d)).

In a number of cases, we observed the simultaneous disruption of several (3–4) spectrin filaments at contact points with ankyrin complexes (red markers, Figure 4(b)).

In other cases, we observed a broken connection between spectrin filaments and the actin complex. The 3D image of a spectrin matrix element in Figure 4(c) shows six distorted connections to actin filaments forming a cone. The diameter of such local craters was estimated as *L* = 300 ± 100 nm. Disruption of the spectrin filaments led, in turn, to a shortening of the distance between membrane proteins, resulting in their clustering (blue markers fused to each other in Figure 4(d)).

Such nanodefects could appear separately or simultaneously; however, the probability of their occurrence was changed with an increase in temperature. The structures shown in Figure 4(a) may be observed under 36–37°С. The topological structures indicated in Figures 4(b)–4(d) were arisen under 39–40°С.

### 3.2. The Nanostructure of the RBC Spectrin Matrix Is Influenced by Temperature

As with other biopolymers, the structural properties of spectrin depend on blood temperature. Here, we investigated the spectrin matrix after incubating blood at a physiological range of temperatures (36–42°С), at much higher temperatures (49–50°C), and at room temperature (20°С).

#### 3.2.1. 20°С

After fresh blood was left in a microvette at 20°C for 30 min, 95 ± 3% of RBCs appeared as discocytes and a regular spectrin network structure was observed. The typical length of spectrin filaments was *S*_20°C_ = 150 ± 60 nm, which coincides with that under control conditions. The elevation difference was *h*_20°C_ = 4 ± 2 nm. Such pattern corresponds to the basic variant of the spectrin matrix.

#### 3.2.2. 36–37°С

Incubation of blood in a thermostat at a physiological temperature of 36–37°C for 30 min did not change the cells' morphology or the structure of the spectrin matrix as compared to the basic variant (Figures 5(a) and 5(b)).

The spectrin network, with its protein complexes and filaments, was clearly visible (Figures 5(b) and 5(c)) and corresponded to the blue, red, and yellow markers in Figure 4(a). The dark zones corresponded to areas of lipid bilayer destruction (holes). In Figure 5(c), red arrows show the structures between which the parameters were measured. On the profile in Figure 5(d), the corresponding zones are marked by dots. The typical size of a hole was *L*_36–37°C_ = 150 ± 60 nm and the height difference *h*_36–37°C_ = 4.2 ± 1.8 nm.

#### 3.2.3. 39–40°С

**Figure 8:**
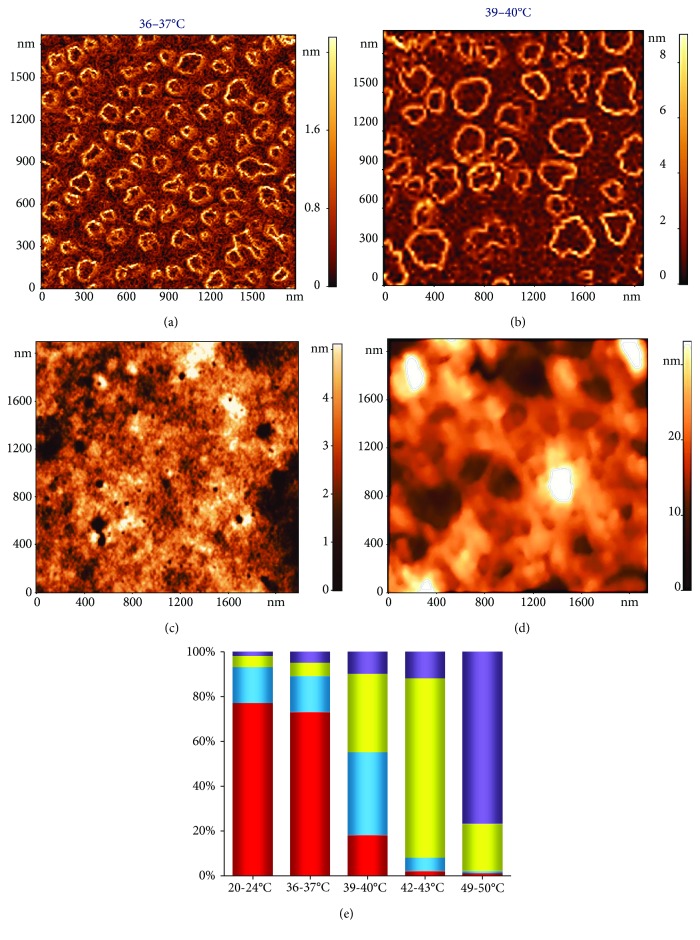
AFM 2D images showing the basic typical variants of the spectrin matrix nanostructure, 2000 × 2000 nm^2^. (a) Regular spectrin network with a typical element < 250 nm (red square). (b) Regular spectrin network with enlarged deep spectrin matrix elements > 250 nm (blue square). (c) Irregular structure (yellow square). (d) Chaotic pattern (purple square). (e) Histogram showing the percentage of each of the structures in (a–d) at various temperatures (20–24°С, 36–37°С, 39–40°С, 42–43°С, and 49–50°С); colours correspond to those in (a–d). Experimental data are shown as mean values.

When blood was incubated at 39–40°C for 30 min (Figure 6(a)), the morphology of the cells changed. Discocytes amounted to 86 ± 8% of cells, echinocytes to 6 ± 1%, and target cells (codocytes) to 8 ± 3% (green arrows, Figure 6). Two spectrin network ensembles were detected: the first (Figures 6(b)–6(d)) corresponded to 37 ± 6% of cases, the second (Figures 6(f)–6(h)) to 35 ± 6% of cases, as indicated by a representative 2D image of the spectrin matrix and a fragment of its nanostructure. In ensemble 1, the spectrin matrix displayed a regular structure (Figures 6(b)–6(d)), but the size of network elements was greater than that observed at 20°C and 36–37°C (Figure 5); specifically, it was calculated as *L*_39–40°C(1)_ = 240 ± 60 nm. 2D images (Figures 5(b) and 5(c)) and the corresponding surface profiles (Figure 6(d)) indicated that the entire actin complex was defective. The local topological nanodefects were in the form of a frustum with height *h*_39–40°C(1)_ = 5.5 ± 1.5 nm.

In some zones of the spectrin network, another type of connection distortion could be detected, namely, discontinuities in the ankyrin complex that resulted in a partial ring structure (Figures 4(b) and 6(c)). The discontinuity of several neighbouring connections at this temperature range was higher than at 36–37°C.

Ensemble 2 displayed an irregular, disordered structure of the spectrin matrix (Figures 6(f)–6(h)), which differed from the ordered network observed previously (Figures 5(b) and 5(c)). In ensemble 2, typical distances showed a wide dispersion, with *L*_39–40°C(2)_ = 220 ± 160 nm and *h*_39–40°C(2)_ = 2.6 ± 1.2 nm.

#### 3.2.4. 42–43°С

A temperature increase to 42–43°С resulted in target cells in the monolayer increasing to 25 ± 8% (Figure 6(e)). The proportion of ensemble 2-type spectrin matrix (Figures 6(f) and 6(g)) also increased to 83 ± 8%. The typical parameters of the spectrin nanostructure were *L*_42–43°C(2)_ = 220 ± 120 nm and *h*_39–40°C(2)_ = 5 ± 3 nm. Moreover, the spectrin matrix was not uniformly irregular across the entire surface of the network. Specifically, it appeared less irregular towards the centre of the cell, in line with the target structure.

#### 3.2.5. 49–50°С

This is the temperature of spectrin denaturation [29]. Total damage to spectrin filaments can lead to alterations in the spectrin matrix as a whole, in turn leading to changes in RBC morphology. As shown in Figure 7(a), the shape of RBCs differed from that at 36–37°C (Figure 5(a)), with discocytes accounting for only 1.0 ± 0.5% of cells.

AFM images of the spectrin network (Figure 7(b)) revealed marked differences compared to the control (Figure 2(c) and 5(b)), with 79 ± 6% of the extracellular surface being strongly damaged. A regularly shaped spectrin matrix was not observed, which also means it was impossible to carry out adequate quantitative estimations of its parameters. A chaotic pattern was observed on the surface (Figure 7(c)), whose typical parameters were *L*_49–50°C_ = 250 ± 150 nm and *h*_49–50°C_ = 15 ± 7 nm (Figure 7(d)). Moreover, protruding structures were observed on the surface, possibly formed by spectrin, proteins, or vesicles.

The shape and geometric parameters of spectrin matrix nanostructures were also estimated based on AFM 2D images reconstructing the gradient distribution of the nanostructure's height over its surface, *grad* *h* (*x*, *y*) (Figure 3). Bright outlines represented fields where the *grad* *h* (*x*, *y*) value was larger. This occurred in regions where the height changed abruptly, such as where discontinuities and dips in the spectrin network were detected. Conversely, dark areas corresponded to regions where *grad* *h* (*x*, *y*) ≈ 0; that is, the height did not change.

Thus, an increase in the temperature from 36 to 50°С resulted in a substantial transformation of the spectrin matrix nanostructure, from a regular spectrin network to an irregular and disordered pattern. The dynamics of representative spectrin matrix nanostructure transformations as a function of increasing temperature are shown in Figure 8. As blood temperature increased, the percentage of different spectrin matrix configurations on the surface of ghost RBCs changed and is summarised by the histogram in Figure 8(e). The colours on the histogram correspond to the markers on the AFM 2D images shown in Figures 8(a)–8(d).

Going from 20 to 50°С, the percentage of spectrin networks whose elements were < 250 nm dropped from 75 ± 10% (20–36°C) to 18 ± 8% (39–40°C) and finally to 1.0 ± 0.3% (49–50°C).

The change in spectrin matrix nanostructure was associated with a disruption of the connections between spectrin filaments and membrane proteins, topological dips and clustering of membrane proteins, and spectrin denaturation. The probability of these processes varied with temperature, which led to a certain dynamics in network configurations.

## 4. Discussion

In the present study, we performed *in vitro* biophysical experiments to show specific topological nanodefects in the spectrin matrix under varying blood temperature conditions. Temperature is the main factor in the study of any material as it affects intermolecular interactions and, thus, *phase transition* of matter. Temperature is particularly important for biological molecules, such as polymers and biopolymers, of which spectrin is an example [30]. A key point addressed by the present work was the influence of temperature in the physiological range (36–42°C) on the spectrin matrix structure.

Spectrin is the main protein of the spectrin matrix; it is made of a fibrillar molecule with a length of 180–200 nm and a thickness of 2–3 nm [31]. The spectrin molecule consists of two large alpha and beta polypeptide chains, associated in the antiparallel direction, and with a length of 100 nm. Dimers are self-associated into tetramers, and these interact with ankyrin. The binding of ankyrin to the cytoplasmic domain enables the attachment of the cytoskeleton to the plasma membrane (Figure 4). At its end, the tetramer binds the protein band 4.1 and the short actin filament, forming a network [32, 33].

Under normal physiological conditions (36–37°C), most of the spectrin matrix nanostructure (>70%) forms a regular network with spacing comparable to the length of spectrin filaments (Figures 5, 8(a), and 8(e)). Higher physiological temperatures (39–40°C) result in a transitional state (Figure 6, 8(b), 8(c), and 8(e)), whereby only 20% of the spectrin matrix forms a regular network and 40% forms an ordered structure but with spacing greater than in the control, and the remaining spectrin matrix is found in an irregular structure that does not resemble a network. The consequence of this distribution may be a change in the cells' morphology and altered blood rheology. Finally, at 42–43°C, almost 95% of the spectrin matrix forms an irregular structure.

Special attention was given to temperature in the range of 49–50°C, at which spectrin denaturation occurs [29]. This leads to the RBC membrane *in vitro* to lose its stability [29]. In this case, the spectrin matrix is in a chaotic state in almost all RBC ghosts (Figure 7, 8(d), and 8(e)), making it impossible to adequately quantify the parameters of the spectrin structure.

All these processes and measurements are of a statistical nature, explaining why at 39–40°C regular networks and irregular structures are observed simultaneously, whereas at 36–37°C most of the spectrin matrix has a regular and periodically spaced structure, and at 49–50°C it acquires a clearly irregular structure.

An increase in temperature can cause rupture of the bonds in the spectrin matrix, akin to that observed following the action of toxic and oxidative factors. Changes to the nanostructure of the spectrin matrix will lead to different outcomes, depending on whether the factor acts on actin [34] or ankyrin junctions [35, 36].

Local alterations to the spectrin matrix arising from changes in blood temperature are potential active centres for local topological defects in RBC membranes. Similar local topological defects were observed following the action of pharmacological agents, ionizing radiation, and long-term storage of blood [15, 23, 25, 37], resulting in alterations to the biophysical processes in RBCs.

A change in the spectrin matrix can alter the rheological properties of blood. Previous studies have investigated the relationship between the mechanical properties of RBCs and the structural basis of the spectrin matrix. For example, they have explored the correlation between the size of the cytoskeleton network and stiffening of the cells [3]. The membrane skeleton is assumed to be responsible for maintaining the shape of the erythrocyte and permitting the large deformations that allow it to survive repeated passage through narrow capillaries [4]. The reversible rearrangement of the spectrin network has been shown to allow rapid and large deformations, thus ensuring the mechanical stability of the membrane [28]. Given their role as structural nodes in the membrane skeleton, actin filaments directly control the biomechanical properties of the RBC membrane [38]. Theoretical studies have also been conducted to establish the relationship between mechanical properties of deformable RBCs and the structure of the spectrin matrix [30]. At the same time, much remains unclear. In particular, the structure of spectrin *in vivo* and the processes underlying its changes when the membrane is deformed are still awaiting clarification [39].

## 5. Conclusion

Using direct *in vitro* biophysical experiments under controlled conditions, we show how the nanostructure of the RBC spectrin matrix changes from a regular network to a chaotic pattern with increasing temperature. This study can be used as the basis for understanding how an increase in body temperature can affect RBC membrane nanostructure, morphology, and, ultimately, blood rheology.

## Data Availability

The data used to support the findings of this study are included within the article.
